# A single low dose of primaquine is safe and sufficient to reduce transmission of *Plasmodium falciparum* gametocytes regardless of cytochrome P450 2D6 enzyme activity in Bagamoyo district, Tanzania

**DOI:** 10.1186/s12936-022-04100-1

**Published:** 2022-03-12

**Authors:** Richard Owden Mwaiswelo, Billy Ngasala, Dominick Msolo, Eliningaya Kweka, Bruno P. Mmbando, Andreas Mårtensson

**Affiliations:** 1 Department of Research and Training, Tropical Pesticides Research Institute, Arusha, Tanzania; 2 Department of Microbiology, Immunology and Parasitology, Hubert Kairuki Memorial University, Dar es Salaam, Tanzania; 3 Department of Medical Parasitology and Entomology, Muhimbili University of Health and Allied Sciences, Dar es Salaam, Tanzania; 4 Department of Women’s and Children’s Health, International Maternal and Child Health (IMCH), Uppsala University, Uppsala, Sweden; 5 College of Natural and Applied Sciences, University of Dar Es Salaam, Dar es Salaam, Tanzania; 6 Tanga Research Centre, National Institute for Medical Research, Tanga, Tanzania

**Keywords:** Safety, Efficacy, Single low-dose primaquine, *Plasmodium falciparum* gametocytes, Cytochrome P450 CYP2D6, G6PD deficiency

## Abstract

**Background:**

Primaquine is a pro-drug and its active metabolite is potent against mature *Plasmodium falciparum* gametocytes. Primaquine is metabolized by a highly polymorphic cytochrome P450 2D6 (CYP2D6) enzyme. Mutations in the gene encoding this enzyme may lead to impaired primaquine activity. This study assessed if 0.25 mg/kg single-dose primaquine is safe and sufficient to reduce transmission of gametocytes in individuals with no, reduced, or increased CYP2D6 enzyme activity.

**Methods:**

Between June 2019 and January 2020 children aged 1–10 years, attending at Yombo dispensary, Bagamoyo district, with confirmed microcopy-determined uncomplicated *P. falciparum* malaria were enrolled in the study. The enrolled patients were treated with a standard artemether-lumefantrine regimen plus 0.25 mg/kg single-dose primaquine and followed up for 28 days for clinical and laboratory assessment. Primaquine was administered with the first dose of artemether-lumefantrine. Safety assessment involved direct questioning and recording of the nature and incidence of clinical signs and symptoms, and measurement of haemoglobin (Hb) concentration. Blood samples collected from 100 patients were used for assessment of post-treatment infectiousness on day 7 using mosquito membrane feeding assays. Molecular methods were used to determine CYP2D6 and glucose-6-phosphate dehydrogenase (G6PD) status. The primary outcome was the safety of 0.25 mg/kg single-dose primaquine based on CYP2D6 status.

**Results:**

In total, 157 children [median age 6.4 (Interquartile range 4.0–8.2) years] were recruited, of whom 21.0% (33/157) and 12.7% (20/157) had reduced CYP2D6 and deficient G6PD activity, respectively. Day 3 mean absolute Hb concentration reduction was 1.50 g/dL [95% confidence interval (CI) 1.10–1.90] and 1.51 g/dL (95% CI 1.31–1.71) in reduced and normal CYP2D6 patients, respectively (*t* = 0.012, *p* = 0.990). The day 3 mean absolute Hb concentration reduction in G6PD deficient, G6PD normal and heterozygous female was 1.82 g/dL (95% CI 1.32–2.32), 1.48 g/dL (95% CI 1.30–1.67) and 1.47 g/dL (95% CI 0.76–2.18), respectively (*F* = 0.838, *p* = 0.435). Sixteen percent (16/98) of the patients each infected at least one mosquito on day 7, and of these, 10.0% (2/20) and 17.9% (14/78) had reduced and normal CYP2D6 enzyme activity, respectively (*x*^*2*^ = 0.736, *p* = 0.513).

**Conclusion:**

Single-dose 0.25 mg/kg primaquine was safe and sufficient for reducing transmission of *P. falciparum* gametocytes regardless of CYP2D6 or G6PD status.

*Trial registration* Study registration number: NCT03352843.

**Supplementary Information:**

The online version contains supplementary material available at 10.1186/s12936-022-04100-1.

## Background

The scale-up of malaria control efforts particularly using insecticide-treated bed nets and artemisinin-based combination therapy (ACT) has led to the global decline of malaria transmission, consequently renewing the interest in the possibility of eliminating the infection [1–3]. Artemisinin derivatives are highly efficacious against both asexual and young *Plasmodium falciparum* gametocytes in many malaria-endemic settings and this has contributed significantly to the global decline of malaria transmission [2, 4]. However, artemisinin derivatives are not potent against mature *P. falciparum* gametocytes, and, therefore, not sufficient to prevent transmission [4].

The emergency of *P. falciparum* resistance against artemisinin in Southeast Asia [5–7], and *Anopheles* resistance against pyrethroid in Africa [8–10], is also threatening the global malaria control efforts hence reinforcing the interest in the use of transmission-blocking drugs to limit the spread of artemisinin resistance and accelerate malaria elimination efforts [1, 11].

Primaquine is an 8-aminoquinoline pro-drug indicated for radical cure of *Plasmodium vivax* and *Plasmodium ovale* infection [12–14]. It is also the only readily available drug potent against mature *P. falciparum* gametocytes, a parasite stage responsible for onward transmission of the infection [12, 15, 16]. The World Health Organization (WHO) recommends the addition of 0.25 mg/kg single-dose primaquine to ACT for the elimination of malaria in low transmission settings and containment in areas threatened by artemisinin resistance [4, 17]. It may also be used in moderate and high malaria transmission settings to reduce transmission [1]. Available data support that a single low-dose (SLD) of primaquine administered with ACT is efficacious for reducing transmission of mature *P. falciparum* gametocytes [18–23], and is also safe even in glucose-6-phosphate dehydrogenase (G6PD) deficiency [23–25]. A failure rate of 0–18.5% against gametocyte clearance and reduction of transmission has however been reported after treatment with even high doses of primaquine, suggesting an involvement of other factors in determining primaquine efficacy [21–23].

Primaquine gametocytocidal effect and haematological toxicity are thought to depend on cytochrome P450 isoenzyme 2D6 (CYP2D6) conversion of a pro-drug into active phenolic metabolites [26–29]. But CYP2D6 is highly polymorphic [29–31], and a single point mutation in a gene encoding for CYP2D6 may lead to either null (CYP2D6*4 and CYP2D6*5), reduced (CYP2D6*17 and CYP*29), or increased enzyme activity (CYP2D6*2) [32, 33], that in turn probably affects the safety and efficacy of primaquine against *P. vivax* and *P. ovale* hypnozoites, and *P. falciparum* transmission [29–31]. The null, reduced, normal, and increased CYP2D6 isoenzyme activity occurs in Africa at an average frequency of 2.8%, 10.6%, 83.4%, and 3.8%, respectively, varying from one ethnic group to another [34, 35]. Reduced CYP2D6 activity is thought to diminish the metabolism of primaquine [28], hence reducing its gametocytocidal effect [36]. However, primaquine sterilizes *P. falciparum* gametocytes probably at a lower concentration than that needed to kill and clear them from the circulation [37]. Of note, molecular methods are commonly used to assess gametocytes carriage following treatment with primaquine [38–40], however, these methods cannot tell whether the detected gametocytes have been sterilized by the drug. However, since sterilization rather than killing and clearance of gametocytes is more important for reducing transmission of the infection, then assessment of infectivity through mosquito membrane feeding assays is an effective method for assessing the efficacy of primaquine than molecular methods. It is not clear if the available primaquine concentration in individuals with reduced CYP2D6 activity can sterilize gametocytes and hence reduce malaria transmission. Conversely, reduced CYP2D6 metabolism probably increases exposure to primaquine parent drug as well as to carboxyprimaquine [28, 31], which probably leads to adverse events (AEs) not normally seen in individuals with normal CYP2D6 [28, 33]. Increased CYP2D6 activity on the other hand, probably increases the risks of AEs due to increased exposure to phenolic metabolites [28]. This study assessed the safety and efficacy of 0.25 mg/kg single-dose primaquine added to artemether-lumefantrine standard regimen for reducing transmission of *P. falciparum* gametocytes in patients with uncomplicated malaria based on CYP2D6 alleles common in African Tanzanian.

## Methods

### Study area

The study was carried out at Yombo primary health facility, Bagamoyo district, Tanzania, between June 2019 and February 2020. The health facility is located southwest, about 20 km from Bagamoyo town. It serves approximately 7000 people and can carry out routine malaria microscopy and rapid diagnostic tests [25]. The inhabitants in the catchment area are predominantly belonging to Kwere and Zaramo ethnic groups. Yombo is a rural area, and the major economic activities are farming, livestock keeping, petty trading, and fishing.

Malaria transmission in the Bagamoyo district occurs throughout the year with peaks corresponding to the long and short rainy seasons from May to July and November to December, respectively. *Plasmodium falciparum* and *Anopheles arabiensis* are the predominant malaria parasite species and vector, respectively [41]. Malaria transmission in the area is moderate with an incidence rate of 69.1 per 1000 population [42]. Artemether-lumefantrine is the first-line treatment for uncomplicated malaria in Tanzania since 2006 [39], and primaquine is now included in the malaria treatment guidelines [44]. Insecticide-treated mosquito bed net is the major vector control method [45]. Previous estimates have shown the prevalence of CYP2D6 normal/increased, reduced and no activity in the area to be 60%, 37%, and 3%, respectively [46].

### Study design

There is no rapid diagnostic test at the moment that could be used to evaluate and group participants based on their CYP2D6 status at the enrolment. Thus this study was a single-arm clinical trial to assess the safety and efficacy of 0.25 mg/kg single-dose primaquine added to standard artemether-lumefantrine treatment in individuals with CYP2D6 reduced or no activity compared to those with normal or increased activity for clearance and reducing transmission of *P. falciparum* gametocytes. Patients with microscopically confirmed uncomplicated *P. falciparum* mono-infection were enrolled in the trial, treated with standard artemether-lumefantrine regimen plus 0.25 mg/kg single-dose primaquine, and then followed-up until day-28 after treatment initiation for clinical and parasitological assessment. Individuals with reduced CYP2D6 activity were defined as those with CYP2D6*17 and CYP*29 alleles, whereas those with no activity were those with CYP2D6*4 and CYP2D6*5, and normal individuals were those with CYP2D6*1, while those with CYP2D6*2 were defined as having increased activity [31, 46]. Additionally, sequencing was performed to identify unreported CYP2D6 point mutations in the study population. A previous study indicated a very low prevalence of poor metabolizers to the extent that would not lead to a significant conclusion [46], therefore, for this study poor metabolizers were combined with those with reduced CYP2D6 activity to form one group, the reduced CYP2D6 activity group.

### Study population, recruitment, and administration of consent

Patients presenting at the health facility with suspected acute uncomplicated malaria were screened for eligibility. Inclusion criteria were age from 1 to 10 years, weight ≥ 10 kg, body temperature ≥ 37.5 °C or history of fever in the last 24 h, microscopy confirmed *P. falciparum* mono-infection, parasitaemia level of 2000–200,000/µL, ability to swallow oral medication, ability and willingness to abide by the study protocol and the stipulated follow-up visits, and a written informed consent from a parent/guardian and assent from children aged ≥ 7 years. Children aged below 1 year were not included in the study because primaquine is contraindicated in this group, and children up to the age of 10 years were included to increase the recruitment rate. Exclusion criteria were evidence of severe malaria or danger signs, known allergy to trial medicines, reported anti-malarial intake ≤ 2 weeks, haemoglobin < 5 g/dL, blood transfusion within last 90 days, febrile condition other than malaria, and known underlying chronic or severe disease (including severe malnutrition).

At the health facility patients and their parents/guardians were informed in detail about the trial, purpose, the risks and benefits of participating in the trial, and their rights, and encouraged them to participate in case they are diagnosed with malaria. Thereafter, patients were evaluated clinically, and those with signs and symptoms of malaria were directed to the laboratory for parasitological evaluation.

For the children who were found to have uncomplicated *P. falciparum* malaria infection, and met all the inclusion and none of the exclusion criteria their parents/guardians were requested to allow their children to participate in the trial. The parents/guardians were then administered with the consent form. The consent form was self-administered to the literate parents/guardians and for illiterate one’s the trial clinician explained to them. All the procedures were done in the presence of a witness to ensure that there is no coercion. Parents/guardians were given time to ask questions to their satisfaction. All consented parents/guardians signed the form either by a written signature for literate or a thumbprint together with a written signature of their witnesses for the illiterate.

### Treatment

Enrolled patients were treated using a standard 3-day course of artemether-lumefantrine (Artefan, Ajanta Pharma Ltd) according to Tanzanian national treatment guidelines for uncomplicated *P. falciparum* malaria [43]. The artemether-lumefantrine dispersible tablets suspended in water were administered to patients who were not able to swallow whole tablets. A single 0.25 mg/kg primaquine dose (Primaquine phosphate, Sanofi) was administered together with the first dose of artemether-lumefantrine [25]. Accuracy of the primaquine dose was ensured by administering primaquine in an aqueous solution. A 15 mg primaquine tablet was suspended in 15 mL of water, and then the dose was measured using a sterile syringe based on the patient’s body weight and weight band. The primaquine dose in an aqueous solution was mixed with glucose-based syrup to mask primaquine bitterness. Biscuits were administered before each dose of artemether-lumefantrine and primaquine to optimize absorption of artemether-lumefantrine and minimize gastrointestinal side effects of primaquine [23, 25]. All the treatment doses were directly observed. A study nurse administered the medicines and observed the participants for 30 min after each dose. A full dose was readministered in case of vomiting within this period. Patients with a fever over 38 °C were treated with paracetamol. Parents/guardians were also instructed to use tepid sponging to lower body temperature in under five years old children.

### Patient withdrawal

Patients were withdrawn from the study in case of vomiting the study drug > 3 times, withdrawal of consent, the onset of a serious febrile illness, intake of any drug with anti-malarial properties outside the study protocol, or any protocol violation. Patients who missed scheduled follow-up visits and did not show up on the successive days despite efforts to trace them at their residences were considered lost to follow-up and were consequently censored from the analysis on the last day they were seen. Participants who returned before the last day of follow-up were not considered as lost to follow-up. Patients with symptoms/signs of severe disease (including repetitive vomiting of study drug) were managed according to the Tanzania national guidelines and followed up until recovery, but were censored from the analysis on the day of withdrawal [39]. Patients with non-severe clinical or parasitological failure after day 14 were re-treated with artemether-lumefantrine.

### Procedures

Clinical and laboratory assessments were performed on days 0, 1, 2, 3, 7, 14, 21, and 28 or any day of recurrent illness as follows:

#### Clinical and safety assessments

The assessment involved taking a history of clinical symptoms, possible adverse events (AEs), concomitant drug consumption, and clinical examination including measurement of axillary temperature. Fever was defined as a body temperature ≥ 37.5 °C. All the clinical and laboratory data were recorded in a case record form (CRF).

Safety assessment for AE and serious adverse events (SAE) was performed using the Primaquine Roll Out Monitoring Pharmacovigilance Tool (PROMPT) [47]. An AE was defined as any unfavorable sign, symptom, syndrome, or disease that developed or worsened during the study even if the event was not considered to be related to the study drug, whereas a SAE was defined as any untoward medical occurrence that was life-threatening; required hospitalization or prolongation of hospitalization; resulted in a persistent or significant disability or incapacity or resulted in death [48]. The AEs were further classified into mild, moderate, severe, and life-threatening as follows: mild—easily tolerated, no or minimal interference with daily activities; moderate-low level of inconvenience, greater than minimal interference with daily activities; severe—interrupts normal daily activities, usually incapacitating; life-threatening—life-threatening consequences, urgent intervention indicated, or death.

The safety assessment involved direct questioning and recording the nature and incidence of AE and/or serious adverse events (SAE). Signs and symptoms such as fatigue, weakness, dizziness, headache, palpitations or allergic drug reactions (rash), diarrhoea, abdominal pain, dizziness, itching, fever, and haemoglobin level (before, during, and after treatment) were recorded and determined to whether they were related to the trial medicine. Patients with AE were kept under observation, and those requiring treatments were treated accordingly. Patients with SAE were admitted and if necessary referred to the Bagamoyo district hospital.

All the patients received an information card with instructions on how to identify signs and symptoms of commonly reported AEs including monitoring of urine colour. In addition, patients were given clear containers and asked to provide urine samples on day 0 before the first drug dose and days 1, 2, and or 3. The urine colour estimation for haemoglobinuria was gauged against the Hillmen colour chart with a colour score ranging from 1 to 10 [49]. A colour score of ≥ 5 was classified as haemoglobinuria to indicate acute haemolysis. Acute haemolysis was defined as a fractional haemoglobin fall of ≥ 25% from the pre-treatment value, or macroscopic haemoglobinuria (Hillmen ≥ 5), or other significant clinical concerns of anaemia or haemolysis.

#### Laboratory assessment

##### Blood sampling

Laboratory assessment involved collection of finger-prick blood samples that were used to measure haemoglobin concentration, prepare the thick film for microscopy to assess presence and density of asexual parasitaemia and gametocytaemia, thin-film for asexual parasite species determination, and also blotted on filter paper (Perkin Elmer 266) for molecular analysis of CYP2D6 and G6PD status. On day 7, 3 mL of venous blood were collected in vacutainers from 100 patients randomly selected using research randomizer software (Wesleyan University, Connecticut, USA) [50] and used for mosquito membrane feeding experiments. Filter papers containing blood samples were labeled, air-dried at room temperature for 3–4 h, packed in individual plastic bags, and then stored in a cabinet at room temperature. At the end of the fieldwork filter papers were transported to Ifakara Health Institute (IHI), Bagamoyo, Tanzania where they were stored at − 80 °C waiting for molecular analysis.

##### Haemoglobin measurements

Haemoglobin concentration was measured using a portable spectrophotometer, HemoCue Hb 201 + (HemoCue AB, Ängelholm Sweden), with a precision of ± 0.3 g/dL. The HemoCue was calibrated every morning using a control cuvette at 16.0 ± 0.3 g/dL according to the manufacturer’s instructions. Anaemia was classified as haemoglobin level < 11 g/dL (mild), < 8 g/dL (moderate) and < 5 g/dL (severe).

##### Malaria microscopy

Thin blood smears were prepared only at enrolment, whereas thick blood smears were prepared at all sampling time points. At the enrolment, two pairs of thin and thick smears were collected, and the first pair was quickly stained using 10% Giemsa for 15 min to accelerate the enrolment of patients, whereas the second pair was stained slowly using 3% Giemsa for 1 h. Thick smears collected during follow-up were stained slowly using 3% Giemsa for 1 h. Asexual and sexual parasites on thick smears were counted against 200 white blood cells (WBC). This number was then multiplied by 40, assuming 8000 leukocytes per microlitre of blood, to gain an approximate parasite density per microlitre of blood. A blood slide was considered negative if no parasite was seen after examining 100 fields. Two, independent microscopists read all the microscopy slides. In case they disagreed on the presence of parasitemia or if the density differed by more than 25%, a third independent reading was performed. In the case of positive versus negative results, a third independent reading was used to confirm the reading of the first two readers.

##### Molecular analysis

The genomic DNA was extracted from 100 μL of dried blood spots on filter papers using Quick-DNA™ Miniprep plus Kit Catalog Nos. D4068 and D4069 [51].

##### CYP2D6 analysis

To detect CYP2D6 point mutations, the extracted DNA was amplified using mass array VeriDose CYP2D6 CNV Panel [52]. The CYP2D6 alleles were then characterized as per activity score as follows: (1) wild-type (normal function) alleles for those that encode for CYP2D6 enzymes that have normal (extensive) metabolic activity, (2) reduced function alleles for those that encode for CYP2D6 enzymes that have less metabolic activity than wild-type (normal function) alleles, and (3) non-functional alleles for those that encode for CYP2D6 enzymes that have little or no metabolic activity. The *5 was used as nomenclature for deleted alleles or nun-functional alleles, whereas if no variant alleles were detected the results were reported as “negative”, or wild-type (normal function) [53].

##### G6PD analysis

The G6PD analysis was performed through amplification of the extracted genomic DNA using a single-round PCR followed by gel electrophoresis. Amplicons were loaded on agarose gel containing GelRed™ (Biotium) and then visualized under ultraviolet- transillumination. The molecular weight of the DNA fragments was sized using Gene Ruler TM 100 bp molecular weight marker. Amplicons with an expected band size of 1061 bp for Exon 3–5 were sequenced using the Sanger sequencer. The interested two most common polymorphisms associated with G6PD deficiency in Africa i.e., A376G (rs1050829 A > G/T > C) and G202A (rs1050828 G > A/C > T) were found in Exon 3–5 after analysis of sequences data by Geneious prime software. The outcomes were classified as follows; For males A was defined as wild-type/normal and A− as hemizygous/deficient G6PD status, whereas for females A−A− was defined as homozygous/deficient, AA− and BA− as heterozygous/intermediate and AA and BA as wild-type/normal G6PD status [54].

### Differentiation of recrudescence from new infection

To differentiate recrudescence from new infection, the extracted DNA from patients classified during the 28 days follow-up period as late clinical failure or late parasitological failure was genotyped using a stepwise genotyping of *P. falciparum* block 3 of merozoite surface protein (MSP) 2, block 2 of MSP 1, and region II (RII) of glutamate-rich protein using a nested PCR method [55]. Briefly, the respective initial amplifications were followed by individual nested PCR reactions using allelic family-specific primers for MSP 1 (K1, MAD20, and RO33) and MSP 2 (FC27 and IC) and semi-nested for RII of Glurp. Amplicons were loaded on agarose gel containing GelRed™ (Biotium, Inc. Hayward, California, USA), separated by electrophoresis, and then visualized under ultraviolet- transillumination (Gel Doc™ (Bio-Rad, Hercules, California, USA). The molecular weight of the DNA fragments was sized using GeneRulerTM 100 bp molecular weight marker (Thermo Fisher Scientific, Waltham, Massachusetts, USA). Alleles in each family were considered the same if fragments size were within 20 base pair intervals. Patients with recurrent parasitaemia but with negative PCR results were considered to have unresolved PCR-adjusted outcomes and were excluded in the final analysis.

#### Infectivity to mosquitoes

Infectivity was assessed using mosquito membrane feeding experiments. Blood samples collected on day 7 from a subset of 100 randomly selected participants were used in the experiment to assess the biological evidence of gametocyte sterilization [56–58]. Briefly, 3 ml of venous blood were drawn into a heparin-containing tube. Within 10 min after taking the blood sample, 500 μL were added to glass feeders and fed to 90 locally reared 4–5 day-old female *Anopheles gambiae *sensu stricto mosquitoes through an artificial membrane. These mosquitoes were starved for a minimum of 5 h before the experiments. The membrane feeder setup was maintained at 37 °C using a temperature-controlled water bath and was run for 15 min. Only fully fed mosquitoes were transferred into a holding cage in the insectary and kept on 10% glucose, at 28 °C for eight days for oocyst maturation, whereas the partially fed and unfed ones were destroyed. A minimum of 25 mosquitoes that survived to this period was dissected in 0.5% mercurochrome, and their midguts were examined using microscopy for oocyst prevalence and density. The proportion of infected mosquitoes and the density of oocysts in infected mosquitoes were determined. An infected mosquito was defined as any mosquito with oocysts detected after dissection. Membrane feeding experiments were performed at the IHI, Bagamoyo, Tanzania.

### Study outcomes

All the evaluated study outcomes were compared between CYP2D6 or G6PD status. The primary outcome was the safety of 0.25 mg/kg single-dose primaquine for reducing transmission of *P. falciparum* gametocytes in patients with uncomplicated *P. falciparum* malaria based on CYP2D6 status defined as mean maximal absolute Hb (g/dL) fall during follow-up (day 0–28). Secondary outcomes included: follow-up day of haemoglobin nadir defined as mean day of follow-up (day 0–28) per group with lowest Hb measurement; maximal percentage fall in haemoglobin defined as the percentage relative change of maximal haemoglobin drop during follow-up (day 0–28) from baseline; percentage of participants with anaemia during the days 0–28 of follow-up; percentage of patients receiving blood transfusion during days 0–28; proportion of patients with urine colour change score ≥ 5 using Hillmen Urine Colour Chart during days 0–28; incidence of serious adverse events by sign, symptom, laboratory parameter during days 0–28; proportion of participants with gastrointestinal symptoms within day 0–7 after taking study drug; proportion of patients with G6PD deficiency; proportion of patients who were infectious to mosquitoes on day 7 determined by direct membrane feeding assay; and proportion of patients with PCR-adjusted adequate clinical and parasitological response (ACPR) by day 28. 

### Ethical considerations

The study was conducted following Good Clinical Practices (GCP), the Declaration of Helsinki, and applicable regulatory requirements in Tanzania. Ethical clearances to conduct the trial were obtained from the Muhimbili University of Health and Allied Sciences Institutional Ethics Review Board and the National Health Research Ethics Review Committee of the National Institute for Medical Research. Permission to import and use primaquine was sought from the Tanzania Medicines and Medical Devices Authority. An independent data and safety monitoring board (DSMB) was established, visited the trial site, and performed the interim analysis of safety data. Written informed consent was obtained from parents/guardians of the participants whereas children aged ≥ 7 years also provided assent before their enrollment. The trial is registered at clinicaltrials.gov with the number NCT03352843.

### Statistical analysis

The study was powered to measure the efficacy of primaquine (0.25 mg/kg primaquine dose) in maximum reduction of gametocytaemia from day one through day 28. A sample size of 155 evaluable subjects was chosen to estimate a prevalence of gametocytes carriage measured by QT-NASBA on day 7 of 10% among the individuals with normal/increased activity compared to 35% in reduced activity at a power of 80% [21]. All the evaluated study outcomes were compared between CYP2D6 or G6PD status.

In this trial, a similar prevalence of CYP2D6 genotypes was assumed to be as described by Wennerholm et al. [46]; 63% normal/increased activity vs 37% reduced activity.

No sample size calculation was performed for the number of patients required for membrane feeding experiments but based on other studies [21–23] 100 patients were involved in the experiments and would provide a reasonable sample to assess infectivity. In each assay, 90 mosquitoes were used as described elsewhere [56, 57].

Data were double entered in an electronic database, cleaned, and analysed using R version 3.2.3 (R Foundation, Vienna, Austria) software. The data were analysed based on intention to treat and per-protocol analyses.

The primary safety end-point, the mean maximal haemoglobin concentration (Hb) reduction during 28 days of follow-up was assessed and compared between the CYP450 2D6 status using independent two paired samples *t*-test. In addition, a one-sample *t*-test was used for comparisons of the mean maximal fall Hb with that reported in Uganda by using 0.4 mg/kg single-dose [21]. Proportions were compared using *Chi-*square or Fisher exact test. Data were censored at the time of withdrawal for patients lost to follow-up, withdrawal of consent, and PCR determined reinfection or uncertain PCR outcome. A *p* < 0.05 was defined as statistically significant.

## Results

### Pre-treatment characteristics of the study participants

**Fig. 1:**
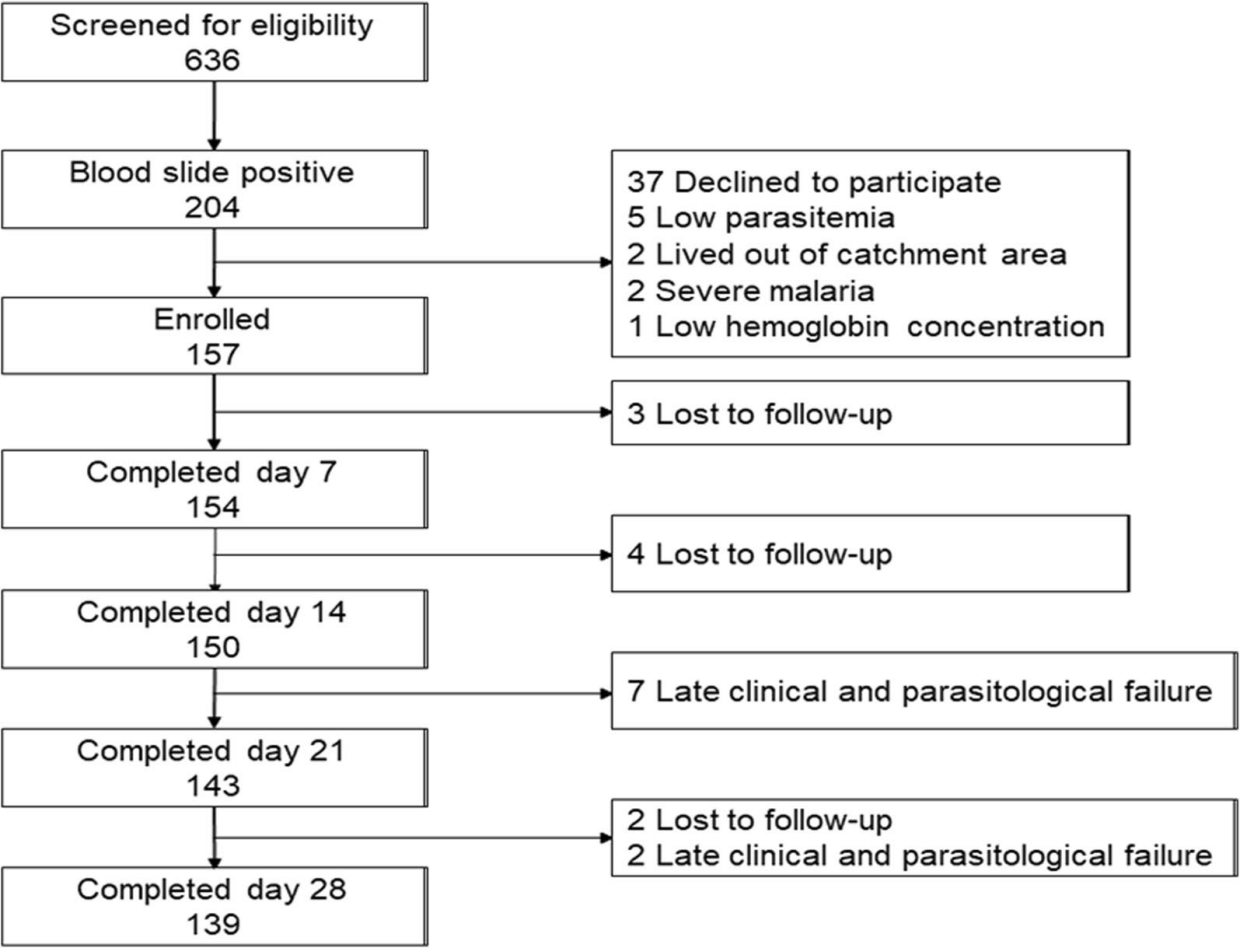
Trial profile of the study participants

A total of 636 patients suspected of having uncomplicated malaria were screened for eligibility to participate in the study, of whom 204 (32.1%) had microscopy-confirmed *P. falciparum* infection. Of the infected patients, 157 were enrolled in the study after fulfilling all the inclusion and none of the exclusion criteria (Fig. 1).

The pre-treatment characteristics of the enrolled patients are presented in Table 1. The prevalence of reduced CYP2D6 enzyme activity among the enrolled patients was 21.0% (33/157) while that of G6PD deficiency was 12.7% (20/157). Only three individuals had increased CYP2D6 enzyme activity, so in the subsequent analyses, this group was combined with the normal CYP2D6 group.

**Table 1:** Pre-treatment characteristics of the study participants

Variable estimate	
Male sex, n (%)	87 (55.4)
Age (years), median (Interquartile range)	6.4 (4.0–8.2)
Weight (kg), mean (SD)	18.9 (5.5)
Axillary temperature (°C), mean (SD)	38.1 (1.2)
Febrile (≥ 37.5 °C), n (%)	109 (69.4)
Hb (g/dL), mean (SD)	10.8 (1.4)
CYP2D6 reduced Hb (g/dL), mean (95%CI)	11.3 (10.8–11.7)
CYP2D6 normal Hb (g/dL), mean (95%CI)	10.6 (10.4–10.9)
CYP2D6 increased Hb (g/dL), mean (95%CI)	11.3 (6.9–15.7)
G6PD deficient Hb (g/dL), mean (95%CI)	10.8 (9.8–11.7)
G6PD normal Hb (g/dL), mean (95%CI)	10.8 (10.5–11.0)
Heterozygous female, Hb (g/dL), mean (95%CI)	10.8 (10.2–11.2)
Anaemic (Hb < 11.0 g/dL), n (%)	85 (54.1)
Asexual parasite density by microscopy/μL, geometric mean (SD)	16,571 (4.9)
Hemizygous/homozygous G6PD deficient, n (%)	20 (12.7)
G6PD heterozygous female, n (%)	21 (13.4)
CYP2D6 reduced activity, n (%)	33 (21.0)
CYP2D6 increased activity, n (%)	3 (1.9)

*Hb* haemoglobin, *SD* standard deviation, *CI* confidence interval

### Fever and parasite clearance

At the enrolment, 69.4% (109/157) of the patients had a fever. Following the initiation of treatment, fever clearance was rapid with only 22.9% (36/157), 3.2% (5/157), and 0.6% (1/157) of the patients remaining febrile on days 1, 2, and 3, respectively. Microscopy-determined asexual parasites decreased to 81.5% (128/157) on day 1 and 13.4% (21/157) on day 2, after the initiation of the treatment, and none of the patients had parasitaemia on day 3. Only one (0.64%) patient had microscopy-determined gametocytes at the enrolment, and these gametocytes were cleared on day 2 after the initiation of treatment. Another patient had gametocytes detected on day 1 after the initiation of treatment, but was not detected again on day 2 and onward.

### Mosquito infectivity

One hundred patients were randomly selected to provide blood samples for mosquito membrane feeding experiments on day 7 after the initiation of treatment, and of these, 98 (98.0%) provided the blood sample. Sixteen out of 98 (16.3%) patients were infectious to at least one mosquito on day 7. Of the infectious patients, 10.0% (2/20) and 17.9% (14/78) had reduced and normal CYP2D6 enzyme activity, respectively, and the difference between the groups was not statistically significant (*Fisher’s test* = 0.736, *p* = 0.513). In addition, there was no statistically significant difference in the infectiousness between children under five years of age (17.6% (6/34) and those aged 5 years and above (15.6% (10/64) (*x*^2^ = 0.066, *p* = 0.797).

### Treatment outcome

Following treatment, 7.7% (11/143) patients had late clinical and parasitological failure, 6.9% (10/143) had a late parasitological failure, and 6.9% (10/143) had PCR adjusted recrudescence. The day 28 artemether-lumefantrine per-protocol PCR-adjusted adequate clinical and parasitological response (ACPR) was 93.0% (133/143), (Table 2). The per intent to treat PCR-adjusted ACPR was 90.0%.

**Table 2:** Treatment outcomes

Outcome	Test, n (%)
Early treatment failure	0
Late clinical and parasitological failure	11 (7.7%)
Late parasitological failure	10 (6.9%)
Crude cure rate by day 28	127 (88.8%)
Undetermined PCR outcome by day 28	5 (3.5%)
PCR determined reinfection by day 28	6 (4.2%)
Recrudescences	10 (6.9%)
PCR adjusted ACPR by day 28	133 (93.0%)

*ACPR* adequate clinical and parasitological response

### Haematological changes during follow-up

**Fig. 2:**
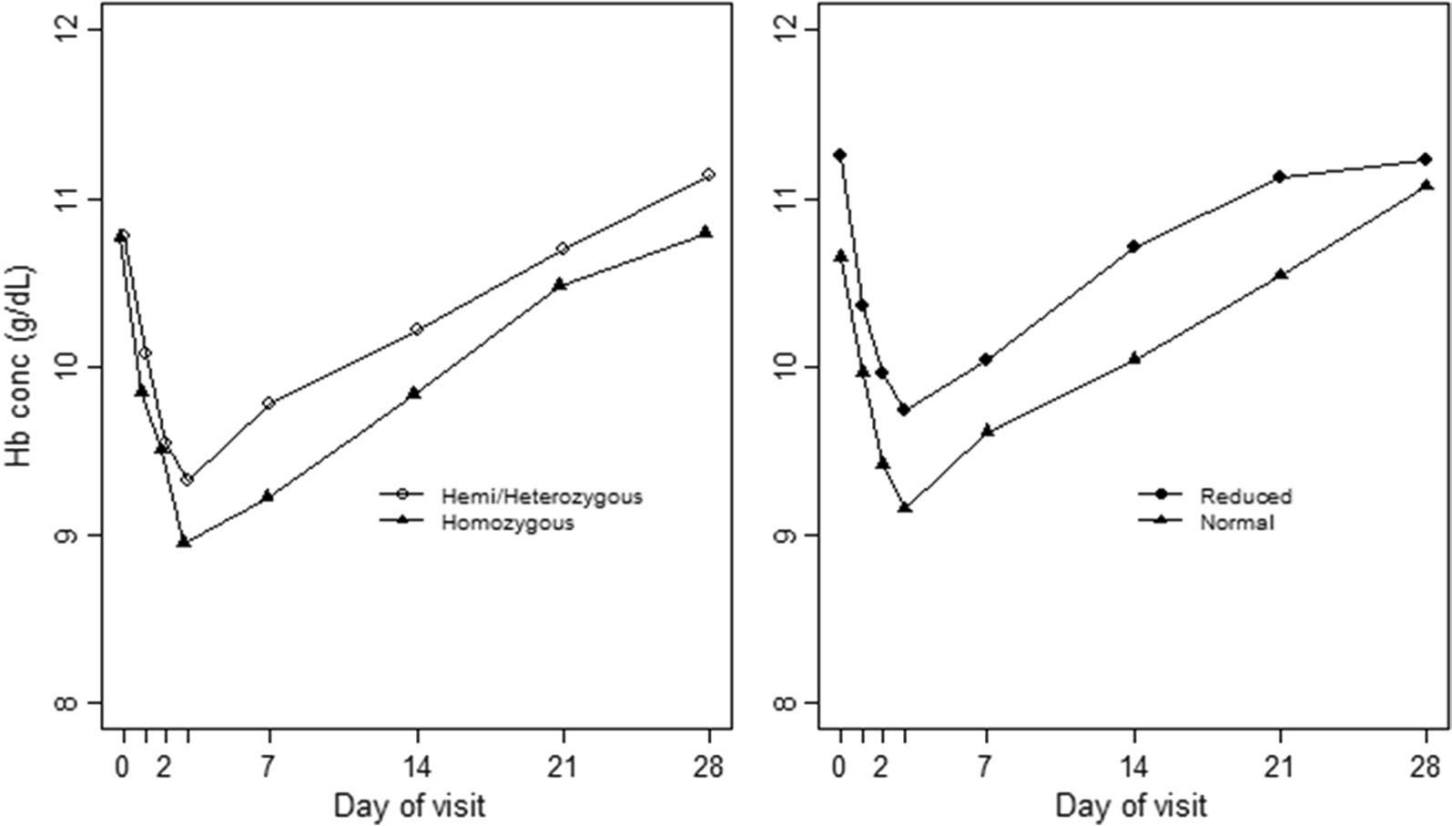
Mean Hb levels across the follow-up time (days) by G6PD (left) and CYP2D6 (right) status

The mean haemoglobin concentration fluctuation during the 28 days of follow-up based on CYP2D6 and G6PD status is presented in Fig. 2. The lowest mean haemoglobin concentration (nadir) occurred on day 3 regardless of CYP2D6 and G6PD status.

The absolute and relative percentage haemoglobin changes between days 0 and 7 are presented in Table 3. There was no statistically significant difference in the mean absolute haemoglobin reduction and mean relative percentage haemoglobin reduction between individuals with reduced and normal CYP2D6 enzyme activity, or between those with G6PD deficiency and G6PD normal or heterozygous females on days 1, 2, 3, and 7. One CYP2D6 and G6PD normal patient had a haemoglobin concentration of 5.9 g/dL at the enrolment and it declined to 4.2 g/dL on day 1 after initiation of treatment, but no blood transfusion ensued. This patient was admitted for two days for observation and recovered.

**Table 3:** Haematological changes between days 0 and 7 based on CYP2D6 and G6PD status

Haematological changes based on CYP2D6 status			
A: absolute haemoglobin reduction			
Day of follow-up	Reduced CYP2D6	Normal CYP2D6	Statistical test
	Mean (95%CI)	Mean (95%CI)	
Day 1	0.86 (0.50–1.22)	0.67 (0.49–0.86)	*t* = 1.034, *p* = 0.303
Day 2	1.28 (0.89–1.65)	1.21 (1.01–1.42)	*t* = 0.383, *p* = 0.703
Day 3	1.50 (1.10–1.90)	1.51 (1.31–1.71)	*t* = 0.012 *p* = 0.990
Day 7	1.16 (0.70–1.62)	1.05 (0.79–1.28)	*t* = 0.428, *p* = 0.669

B: relative % haemoglobin reduction			
Day of follow-up			
Day 1	7.65 (4.24–10.62)	6.12 (4.16–7.55)	*t* = 0.834, *p* = 0.406
Day 2	11.15 (7.84–14.13)	11.02 (8.97–12.64)	*t* = 0.069, *p* = 0.945
Day 3	13.12 (9.51–16.44)	13.80 (11.81–15.43)	*t* = − 0.347, *p* = 0.729
Day 7	9.82 (5.96–13.68)	9.19 (6.86–11.30)	*t* = 0.270, *p* = 0.788

Haematological changes based on G6PD status				
A: mean absolute haemoglobin reduction				
Day of follow-up	G6PD deficient, mean (95%CI)	G6PD Normal, mean (95%CI)	G6PD Heterozygous mean (95%CI)	Statistical Test
Day 1	0.94 (0.52–1.35)	0.69 (0.52–0.88)	0.72 (0.16–1.29)	*F* = *0.*463*, p* = 0.630
Day 2	1.27 (0.83–1.71)	1.24 (1.03–1.45)	1.19 (0.66–1.72)	*F* = 0.028*, p* = 0.972
Day 3	1.82 (1.32–2.32)	1.48 (1.30–1.67)	1.47 (0.76–2.18)	*F* = 0.838*, p* = 0.435
Day 7	1.55 (1.02–2.08)	1.05 (0.82–1.28)	0.75 (− 0.04 to 1.53)	*F* = 2.063*, p* = 0.131

B: mean relative % haemoglobin reduction				
Day of follow-up				
Day 1	8.35 (4.58–12.12)	6.12 (4.44–7.81)	6.43 (1.20–11.66)	*F* = 0.438, *p* = 0.618
Day 2	11.13 (7.28–14.98)	11.07 (9.25–12.89)	10.82 (5.90–15.73)	*F* = 0.007, *p* = 0.993
Day 3	16.46 (12.0–20.92)	13.27 (11.63–14.9)	13.10 (6.39–19.81)	*F* = 0.928, *p* = 0.398
Day 7	13.57 (9.39–17.75)	9.06 (6.95–11.17)	6.57 (− 0.87 to 14.01)	*F* = 1.896, *p* = 0.154

*t* T-test, *F* ANOVA

Furthermore, during follow-up, acute haemolysis defined as haemoglobin reduction of ≥ 25% occurred in 21.7% (34/157) of the patients. Of the patients with acute haemolysis, 18.2% (6/33) and 22.6% (28/124) had reduced and normal CYP2D6 activity, respectively (*x*^*2*^ = 0.297, *p* = 0.586), whereas, 25.0% (5/20), 20.7% (24/116) and 23.8% (5/21) of them were G6PD deficient, normal and heterozygous females, respectively (trend *x*^*2*^ = 0.252, *p* = 0.881).

On the other hand, 45.0% (9/20) of G6PD deficient patients had reduced CYP2D6 enzyme activity. Out of 11 participants with G6PD deficiency and normal CYP2D6 enzyme activity, 5 (45.5%) had acute haemolysis, whereas none (0/9) of participants with G6PD deficiency and reduced CYP2D6 had acute haemolysis (*Fishers’* test, *p* = 0.038).

At the enrolment 39.4% (13/33) and 58.1% (72/124) of patients with reduced and normal CYP2D6 enzyme activities had anaemia, but the prevalence of anaemia was not significantly different between the status (*x*^*2*^ = 3.659, *p* = 0.056). Likewise, the prevalence of anaemia at the enrolment was 55.0% (11/20), 53.4% (62/116), and 57.1% (12/21) in G6PD deficient, G6PD normal, and heterozygous patients, and was not significantly different between the status (trend *x*^2^ = 0.105, *p* = 0.949). During follow-up, the prevalence of anaemia increased significantly from 54.1% (85/157) at the enrolment to 89.8% (141/157) on day 3 (*McNemar* test, *p* < 0.001) and 82.8% (130/157) on day 7 (*McNemar* test, *p* < 0.001). Prevalence of anaemia was not significantly different between CYP2D6 status on days 3 (*x*^2^ = 0.170, *p* = 0.747) and day 7 (*x*^*2*^ = 1.456, *p* = 0.228), but was significantly different between G6PD status with higher prevalence occurring in G6PD normal patients on day 3 (93.1% (108/116), trend *x*^2^ = 6.136, *p* = 0.047) and in G6PD deficient patients (100% (20/20), trend *x*^2^ = 7.994, *p* = 0.018) on day 7.

About 5.7% (9/157) of the patients were both reduced CYP2D6 and G6PD deficient. The haematological changes in patients with both reduced CYP2D6 and G6PD deficiency compared to other CYP2D6 and G6PD status are presented in Table 4. There was no statistically significant difference in mean absolute and relative percentage haemoglobin concentration reduction between CYP2D6 or G6PD status. Although not statistically significant, G6PD deficient patients had a trend of having large values of mean absolute and relative percentage haemoglobin concentration reduction than patients of another status. The prevalence of anaemia was not significantly different between groups on day 3 but was significantly different on day 7 with all the patients with G6PD deficiency and those with both reduced CYP2D6 and G6PD deficiency being anaemic.

**Table 4:** Haematological changes based on CYP2D6 and G6PD status

	Normal for CYP2D6 and G6PD status	Reduced CYP2D6 only	G6PD deficiency only	Both reduced CYP2D6 and G6PD deficiency	Test, *p*-value
	Mean, 95% CI	Mean, 95% CI	Mean, 95% CI	Mean, 95% CI	
Mean absolute Hb reduction					
Day of follow-up					
Day 1	0.64 (0.45–0.83)	0.99 (0.53–1.46)	1.19 (0.55–1.83)	0.62 (0.05–1.19)	*F* = 1.661, *p* = 0.178
Day 2	1.19 (0.99–1.41)	1.40 (0.91–1.88)	1.45 (0.69–2.20)	1.06 (0.56–1.56)	*F* = 0.422, *p* = 0.737
Day 3	1.47 (1.27–1.67)	1.55 (1.04–2.07)	2.12 (1.27–2.97)	1.46 (0.92–1.99)	*F* = 1.214, *p* = 0.307
Day 7	0.96 (0.72)	1.18 (0.58–1.79)	1.92 (1.11–2.72)	1.10 (0.41–1.79)	*F* = 1.932, *p* = 0.127
Mean relative% Hb reduction					
Day of follow-up					
Day 1	5.73 (3.95–7.51)	8.23 (4.35–12.11)	10.17 (4.80–15.55)	6.12 (-0.02–12.26)	*F* = 1.102, *p* = 350
Day 2	10.89 (8.99–12.78)	11.70 (7.63–15.78)	12.34 (5.51–19.13)	9.68 (5.61–13.75)	*F* = 0.165, *p* = 0.920
Day 3	13.30 (11.46–15.13)	12.98 (8.48–17.48)	18.87 (11.20–26.55)	13.51 (8.99–18.02)	*F* = 1.113, *p* = 0.346
Day 7	8.45 (6.15–10.73)	9.79 (4.62–14.96)	16.56 (10.25–22.86)	9.91 (4.37–15.45)	*F* = 1.624, *p* = 0.186
Anaemic					
Day of follow-up					
Day 3, n (%)	91.2% (103/113)	87.5% (21/24)	81.8% (9/11)	88.9% (8/9)	*X*^2^ = 1.138, *p* = 0.768
Day 7, n (%)	83.2% (94/113)	66.7% (16/24)	100.0% (11/11)	100.0% (9/9)	*X*^*2*^ = 8.55, *p* = 0.036

*F* ANOVA, *x*^2^ Chi-square

### Other adverse events

Other adverse events (AEs) occurred in 65.6% (103/157) of the patients. In total 124 AEs were reported during follow-up and were all mild and self-limiting, Table 5. Haemoglobinuria representing acute haemolysis occurred in 11.3% (14/124) of the patients. Of the patients with haemoglobinuria, 3.0% (1/33) had reduced CYP2D6 activity, and 5.0% (1/20) had G6PD deficiency, and the difference in the prevalence between CYP2D6 (*x*^*2*^ = 1.29, *p* = 0.257) or G6PD (*x*^*2*^ = 0.10, *p* = 0.751) status was not statistically significant. Likewise, all other AEs were not statistically significantly different between the CYP2D6 or G6PD status.

**Table 5:** Adverse events reported during follow-up

Adverse effect	Overall	CYP2D6		Test, *p*-value	G6PD		Test, *p*-value
		Reduced	Normal		Deficient	Normal	
Mild urine colour change, n (%)	79 (63.7)	17 (51.5)	62 (50.0)	1.861, 0.394	12 (60.0)	58 (50.0)	1.408, 0.843
Haemoglobinuria, n (%)	14 (11.3)	1 (3.0)	13 (10.5)	1.861, 0.394	1 (5.0)	11 (9.5)	1.408, 0.843
Abdominal pain, n (%)	9 (7.3)	0 (0)	9 (7.3)	2.541, 0.206	2 (10.0)	4 (3.4)	4.637, 0.098
Diarrhoea, n (%)	9 (7.3)	1 (3.0)	8 (6.5)	0.565, 0.686	1 (5.0)	7 (6.0)	0.076, 0.963
Vomiting, n (%)	5 (4.0)	1 (3.0)	4 (3.2)	0.003, 1.0	2 (10.0)	3 (2.6)	3.838, 0.147
Nausea, n (%)	4 (3.2)	1 (3.0)	3 (2.4)	0.039, 1.0	2 (10.0)	2 (1.7)	5.339, 0.069
Dizziness, n (%)	2 (1.6)	0 (0)	2 (1.6)	0.539, 1.0	0 (0)	2 (1.7)	0.716, 0.699
Coughing, n (%)	1 (0.8)	0 (0)	1 (0.8)	0.268, 1.0	0 (0)	1 (0.9)	0.356, 0.837
Rashes, n (%)	1 (0.8)	0 (0)	1 (0.8)	0.268, 1.0	0 (0)	1 (0.9)	0.356, 0.837

## Discussion

Primaquine is the only approved anti-malarial drug potent against mature *P. falciparum* gametocytes [12, 16, 59, 60]. The drug is, however, metabolized by the CYP2D6 enzyme to its active metabolite, and previous studies have indicated the safety and efficacy of primaquine for reducing transmission of *P. falciparum* gametocytes, to probably be influenced by the polymorphic nature of the gene encoding this enzyme [26, 28, 29, 36, 61]. Primaquine use is also limited especially in Africa due to safety concerns particularly in individuals with G6PD deficiency, a condition with a prevalence of up to 33% in the continent [16, 60, 62, 63]. This study, therefore, aimed to assess the safety and efficacy of the SLD of primaquine as assessed by mosquito membrane feeding assays, based on CYP2D6 enzyme activity. Safety was also assessed based on G6PD status. Findings in this study showed that the SLD of primaquine was safe and well-tolerated regardless of CYP2D6 or G6PD status. The mean absolute and relative percentage haemoglobin reduction on days 3 and 7 after the initiation of treatment were not statistically significantly different between CYP2D6 or G6PD status. Similar findings have been reported in previous studies assessing the safety of primaquine based on CYP2D6 [61] or G6PD [23, 25, 64] status. Other studies have indicated a significant haemoglobin reduction in G6PD deficient than normal patients [24, 65, 66]. In this study, the nadir occurred on day 3 regardless of CYP2D6 or G6PD status. Previous studies have also reported similar findings in G6PD deficient versus normal patients following treatment with either ACT alone or plus the SLD primaquine [21, 25, 67]. Furthermore, acute haemolysis defined as haemoglobin reduction of ≥ 25% occurred in about 22% of the patients whereas that indicated by haemoglobinuria/red urine (urine colour change score ≥ 5 on Hillmen scale) occurred in 11.3% of the patients. However, the prevalence of acute haemolysis indicated both by haemoglobin drop of ≥ 25% or haemoglobinuria was not significantly different between CYP2D6 or G6PD status. Conversely, more than half of the patients had anaemia at the enrolment, and the prevalence of the condition increased significantly on day 3, then declined slightly on day 7, but was still significantly higher than that at the enrolment. However, the prevalence of patients with anaemia at the enrolment and on days 3 and 7 following the initiation of treatment were not statistically significantly different between the CYP2D6 status. Nonetheless, there was a significant difference in the prevalence of anaemia among G6PD status on days 3 and 7, with more G6PD normal patients having anaemia on day 3 whereas on day 7 more of G6PD deficient had anaemia. On the contrary, a study in South Africa found a significant increase in the prevalence of anaemia during follow-up only in G6PD normal patients [66]. Of note, in this study, it was hypothesized that reduced CYP2D6 activity would probably compromise primaquine safety due to the accumulation of the parent drug, and it would also be the case in patients with increased CYP2D6 activity as a result of increased production of phenolic metabolites that are implicated in most of primaquine related adverse events. However, in this study, the lack of statistically significant differences in haematological indices including the mean maximal absolute and relative percentage haemoglobin reduction between CYP2D6 or G6PD status during follow-up may probably indicate that SLD primaquine did not cause noticeable haemolysis in the study population and the observed haemoglobin reduction might rather be due to haemolysis caused by malaria infection. Malaria parasites cause haemolysis of infected and non-infected erythrocytes leading to anaemia [68–70]. In addition, major none-haemolytic adverse events reported during follow-up included abdominal pain, diarrhoea, and vomiting, and there was no significant difference in the prevalence of these adverse events between the CYP2D6 or G6PD status. Importantly, similar adverse events have been reported in previous studies following treatment with ACT alone or plus SLD primaquine [23, 25, 65, 66].

Reduced CYP2D6 activity is thought to reduce primaquine efficacy probably by impairing the production of phenolic metabolites that determines the drug’s efficacy [36, 61]. The assumption in this study was that the available primaquine concentration produced in reduced CYP2D6 is sufficient to reduce the transmission of *P. falciparum* gametocytes. Following mosquito membrane feeding assay, 16.3% of the patients were infectious to at least one mosquito on day 7. However, there was no statistically significant difference in the prevalence of infectiousness between CYP2D6 status. In addition, there was no statistically significant differences in the rate of infectiousness between the underfives and those aged above five years. Contrarily, a previous study has reported a higher prevalence of gametocytes carriage in CYP2D6 poor/reduced activity [61]. However, whereas the study by Pett et al. assessed gametocytes clearance using a molecular method [61], this study assessed gametocytes sterilization following primaquine treatment through assessment of infectivity to *Anopheles* mosquitoes. Importantly, sterilization of *P. falciparum* gametocytes occurs at a lower primaquine concentration and earlier than it is required for them to get killed and cleared from the circulation [37], therefore, the gametocytes detected by the molecular method in the previous study might have already been sterilized. Indeed, the absence of significant difference in the prevalence of infectious patients between CYP2D6 status in this study may probably indicate that: first, the primaquine concentration available in individuals with reduced CYP2D6 activity may be sufficient to reduce *P. falciparum* gametocytes transmission; second, other factors such as poor intestinal absorption especially during the acute clinical malaria infection may be responsible for the failure in reducing the transmission; and third, the CYP2D6 reduced activity has a more significant role in *P. vivax* hypnozoites clearance rather than *P. falciparum* gametocytes sterilization.

Rapid fever and asexual *P. falciparum* parasite clearance is a hallmark of artemisinins [71]. In this study fever clearance was rapid. However, microscopy-determined asexual parasite clearance was slow with a three-quarter of patients having parasites on day 1, and nearly a quarter of them had on day 2. However, none of the patients had parasites on day 3. Artemether-lumefantrine had the efficacy of 90% as per intent to treat, and 93.0% as per protocol. Previous studies in the same study area [72, 73], other parts of Tanzania [74–76], and other countries [77–79] using artemether-lumefantrine alone or with the SLD of primaquine have reported slightly higher cure rates. The slow parasite clearance observed especially on day 1 and slightly lower efficacy of 90% as per intent to treat highlights the need for further assessment of the rate of parasite clearance especially using artemether-lumefantrine alone and selection of *P. falciparum* K13 gene.

## Limitations

Despite its strength, the study had limitations including, at the enrolment patients were not tested and then grouped based on the CYP450 2D6 status as there is no rapid test for the condition, thus it was not possible to recruit an equal number of participants based on their CYP2D6 status, and therefore, it was not possible to have a double-blinded randomized clinical trial with artemether-lumefantrine alone versus artemether-lumefantrine plus SLD primaquine. However, the attrition rate of 20% was used to ensure that the required proportions of patients with CYP2D6 normal/increased and reduced/null are recruited. Another limitation is that the sample size calculation was based on previous studies that assessed PQ efficacy for reducing gametocytes transmission using QT-NASBA, but during the implementation, the efficacy was assessed using mosquito membrane feeding assays only, and the study was not powered to assess efficacy using membrane feeding assay as the primary outcome. Furthermore, the available primaquine concentration in the plasma of treated patients was not measured, therefore, its relationship with sterilization of *P. falciparum* gametocytes was not assessed.

## Conclusion

The SLD primaquine was safe and sufficient for reducing transmission of *P. falciparum* gametocytes, as assessed by mosquito membrane feeding assays, regardless of the CYP2D6 or G6PD status, and the observed adverse events were mild and self-limiting.

## Supplementary Information

**Additional file 1.** Dataset.

## Data Availability

All relevant data are within the manuscript and its Additional file 1.

## Abbreviations

ACT: Artemisinin-based combination therapy
AE: Adverse event
CRF: Case record form
DSMB: Data safety monitoring board
EDCTP: European and Developing Countries Clinical Trials Partnership
G6PD: Glucose-6-phosphate dehydrogenase
NIMR: National Institute for Medical Research
mRNA: Messenger ribonucleic acid
PCR: Polymerase chain reaction
PROMPT: Primaquine roll out monitoring pharmacovigilance tool
QT-NASBA: Quantitative nucleic acid sequence based assay
SAE: Serious adverse event
WBC: White blood cells
WHO: World Health Organization
